# Evaluation of Penicillin Allergies and an Allergy Assessment Pilot in the Emergency Department

**DOI:** 10.1017/ash.2021.72

**Published:** 2021-07-29

**Authors:** Ashlyn Norris, Kalynn Northam, Lindsay Daniels, Mildred Kwan, Gary Burke, Nikolaos Mavrogiorgos, Renae Boerneke

## Abstract

Penicillin (PCN) allergy is one of the most frequently reported medication allergies, with ~10% of the US population reporting a PCN allergy. However, studies have shown that only 1% of the US population have a true IgE-mediated reaction to PCN. Delabeling and appropriately updating patient allergy profiles could decrease the use of alternative broad-spectrum antibiotics, rates of infectious complications [*C. difficile*, methicillin-resistant *Staphylococcus aureus* (MRSA), vancomycin-resistant *Enterococcus* (VRE)], antibiotic resistance, and overall healthcare cost. The emergency department (ED) is an important setting in which to assess PCN allergies and to delabel patients when appropriate because there are >130 million ED visits in the United States each year. We sought to determine the percentage of PCN allergy–labeled patients who could be delabeled through a PCN allergy assessment interview in an ED. Key secondary outcomes included the percentage of interviewed patients who could not be delabeled based on history alone but would be eligible for an amoxicillin oral challenge or a PCN skin test (PST). A prospective PCN allergy assessment pilot was performed for patients aged >18 years presenting to the UNC Medical Center ED between December 1 and December 17, 2020, with a documented PCN allergy. A pharmacist conducted penicillin allergy assessments on a convenience sample of patients presenting to the ED between 8 a.m. and 3 p.m. on weekdays. Based on patients’ reported and documented histories, charts were updated with the most accurate information and allergies were delabeled if appropriate. In total, 95 patients were assessed; 62 (65.3%) were interviewed and 15 (24.2%) were delabeled. In addition, 26 patients (41.9%) were deemed eligible for an oral amoxicillin challenge, 19 (30.6%) qualified for a PST, and 2 (3.2%) patients did not qualify for further assessment due to having a an IgE-mediated reaction in the past 5 years. Of the 15 patients who were delabeled, 6 (40.0%) received antibiotics during their admission: 4 (73.3%) of those patients received a penicillin and 2 (36.7%) received a cephalosporin, all without adverse reactions. Patient assessments took ~20 minutes to complete, including chart review, patient interview, and postinterview chart updating. The results from this pilot study demonstrate the impact of performing PCN allergy assessments in ED. Interdisciplinary opportunities should be explored to develop processes that will improve the efficiency and sustainability of PCN allergy assessments within the ED to allow this important stewardship intervention to continue.

**Funding:** No

**Disclosures:** None

